# The Sensitivity of Diffuse Large B-Cell Lymphoma Cell Lines to Histone Deacetylase Inhibitor-Induced Apoptosis Is Modulated by BCL-2 Family Protein Activity

**DOI:** 10.1371/journal.pone.0062822

**Published:** 2013-05-07

**Authors:** Ryan C. Thompson, Iosif Vardinogiannis, Thomas D. Gilmore

**Affiliations:** Department of Biology, Boston University, Boston, Massachusetts, United States of America; Cincinnati Children's Hospital Medical Center, United States of America

## Abstract

**Background:**

Diffuse large B-cell lymphoma (DLBCL) is a genetically heterogeneous disease and this variation can often be used to explain the response of individual patients to chemotherapy. One cancer therapeutic approach currently in clinical trials uses histone deacetylase inhibitors (HDACi’s) as monotherapy or in combination with other agents.

**Methodology/Principal Findings:**

We have used a variety of cell-based and molecular/biochemical assays to show that two pan-HDAC inhibitors, trichostatin A and vorinostat, induce apoptosis in seven of eight human DLBCL cell lines. Consistent with previous reports implicating the BCL-2 family in regulating HDACi-induced apoptosis, ectopic over-expression of anti-apoptotic proteins BCL-2 and BCL-XL or pro-apoptotic protein BIM in these cell lines conferred further resistance or sensitivity, respectively, to HDACi treatment. Additionally, BCL-2 family antgonist ABT-737 increased the sensitivity of several DLBCL cell lines to vorinostat-induced apoptosis, including one cell line (SUDHL6) that is resistant to vorinostat alone. Moreover, two variants of the HDACi-sensitive SUDHL4 cell line that have decreased sensitivity to vorinostat showed up-regulation of BCL-2 family anti-apoptotic proteins such as BCL-XL and MCL-1, as well as decreased sensitivity to ABT-737. These results suggest that the regulation and overall balance of anti- to pro-apoptotic BCL-2 family protein expression is important in defining the sensitivity of DLBCL to HDACi-induced apoptosis. However, the sensitivity of DLBCL cell lines to HDACi treatment does not correlate with expression of any individual BCL-2 family member.

**Conclusions/Significance:**

These studies indicate that the sensitivity of DLBCL to treatment with HDACi’s is dependent on the complex regulation of BCL-2 family members and that BCL-2 antagonists may enhance the response of a subset of DLBCL patients to HDACi treatment.

## Introduction

Diffuse large B-cell lymphoma (DLBCL) is the most common form of lymphoma, accounting for 40% of non-Hodgkin lymphomas and 30% of all lymphomas [1]. Gene expression arrays have revealed distinct DLBCL subtypes that differ in their response to the standard antibody/chemotherapy regimen, R-CHOP [2], [3]. Nevertheless, there is a need for the identification of additional predictive gene expression bio-signatures, in part because many patients do not respond to R-CHOP therapy and because there are a number of new chemotherapeutic approaches being evaluated [4].

One class of therapeutic agents currently in clinical trials includes epigenetic modifiers, mainly histone deacetylase inhibitors (HDACi’s) and DNA methyltrasferase inhibitors. HDACs comprise a family of proteins that deacetylate a variety of protein targets, generally ones involved in transcriptional control [5], [6]. HDACi’s have been shown to be effective at inducing cell death in cancers on their own and in conjunction with other drugs, both in cell lines and in patients [5]–[7]. For instance, vorinostat and valproic acid induce apoptosis in human lymphoid cancers, which is associated with cell cycle arrest [8], [9]. Vorinostat was approved for treatment of T-cell lymphoma [10], and is currently in clinical trials for the treatment of a variety of B-cell lymphomas, showing promising results for certain advanced hematologic malignancies [11], but not for patients with relapsed DLBCL [10]. Additionally, vorinostat has been shown to synergize with the proteasome inhibitors bortezomib in multiple myeloma and carfilzomib in DLBCL [5], [12], with the BH3 mimetic ABT-737 in breast cancer and in certain transgenic murine lymphomas [7], [13], and with the PKCβ inhibitor enzastaurin in DLBCL and T-cell lymphoma [9].

The BCL-2 protein family plays a pivotal role in regulating mitochondrial-derived apoptosis in normal and malignant cell types. The BCL-2 family can be divided into three classes: anti-apoptotic (BCL-2, BCL-XL, MCL-1, A1, BCL-W, BCL-B), BH3-only pro-apoptotic modulators of apoptosis (BIM, BID, PUMA, BIK, BAD, NOXA, BMF), and pro-apoptotic activators (BAK, BAX, BOK) [14]–[16]. BCL-2 family proteins act as regulators of cell survival in a variety of cancers, including non-small cell lung cancer and breast cancer [17], [18], colon adenocarcinomas [19], clear-cell renal cell carcinoma [20], non-Hodgkin B-cell lymphoma [21], and other hematopoietic malignancies [22]. Two examples of BCL-2 misregulation are the occurrence of the *BCL-2* gene as part of the t(14;18) translocation found in a number of non-Hodgkin B-cell lymphomas [23] and the increased expression of BCL-2 in different cancers, in which elevated levels correlate with chemoresistance [24]. High BCL-2 expression, regardless of microarray-based classification, has also recently been reported to define a subset of DLBCL patients with a clinically superior outcome in response to R-CHOP therapy [25].

It is well-established that anti-apoptotic proteins such as BCL-2, BCL-XL, and MCL-1 can sequester multiple pro-apoptotic proteins including BIM and BAX to inhibit apoptosis in several cancer types [16], [18], [25], [26]. While HDACi-induced apoptosis has been shown to occur via up-regulation of the pro-apoptotic BH3-only protein BIM [5], [27], many tumor cells are protected from apoptosis-inducing agents by having increased expression of anti-apoptotic proteins or decreased expression of pro-apoptotic proteins. The interaction between anti-apoptotic and pro-apoptotic proteins has been a target of therapeutic discovery, yielding the BAD-like BH3 mimetic ABT-737 [26], which specifically targets the BH3 binding pocket of BCL-2, BCL-XL, and BCL-W, thereby inhibiting binding of BH3-only modulators and pro-apoptotic activators [14], [15], [27], [28]. ABT-737 has been shown to increase cell death in several cancer types as a monotherapy or in combination with other compounds [13], [29]–[34]. Relevant to this study, ABT-737 has been investigated as part of a combination therapy with vorinostat [7] and bortezomib [35] in multiple myeloma and in a variety of lymphomas and leukemias, respectively. However, the precise interplay between BCL-2 family proteins and sensitivity to HDACi’s in DLBCL has not been fully characterized.

Based on those studies, as well as others from our lab [36], we hypothesized that BCL-2 family member expression could play a role in affecting the sensitivity of DLBCL to HDACi’s that induce apoptosis. In this work, we show that many common DLBCL cell lines are susceptible to HDACi-induced apoptosis through a caspase-dependent mechanism and that exogenous expression of specific BCL-2 family members or treatment with ABT-737 can alter DLBCL cell line sensitivity to HDACi treatment. Moreover, the analysis of two vorinostat-resistant variants of the HDACi-sensitive cell line SUDHL4 revealed that HDACi resistance is correlated with increased expression of BCL-2 family anti-apoptotic proteins.

## Results

### Inhibition of HDAC Activity Induces Apoptosis in the Majority of Cell Lines from a Select Panel of Diffuse Large B-cell Lymphoma Cell Lines

**Figure 1:**
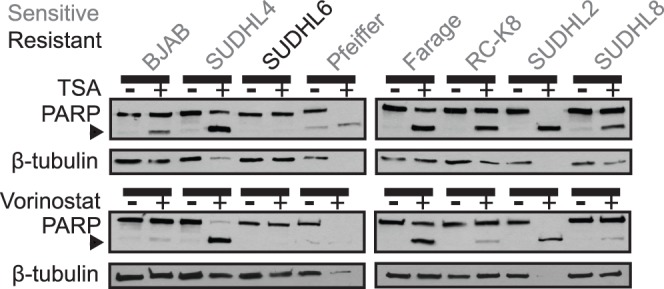
Trichostatin A and vorinostat induce PARP cleavage in a subset of DLBCL cell lines. The indicated DLBCL cell lines were treated with either 300 nM TSA (top panel) or 3 µM vorinostat (bottom panel) for 24 h and Western blotting was performed for PARP and β-tubulin (as a normalizing control). Note: in cases where cells undergo extensive PARP cleavage (e.g., SUDHL4, Pfeiffer, SUDHL2), tubulin can appear under-loaded (likely because tubulin also begins to be degraded in these dying cells). Cell lines used were GCB-like (BJAB, SUDHL4, SUDHL6, Pfeiffer, Farage, and SUDHL8) and ABC-like (RC-K8 and SUDHL2). Grey font indicates HDACi-sensitive cell lines; black font indicates HDACi-resistant cell line SUDHL6. The arrowhead indicates cleaved PARP.

As a first assessment of the ability of HDACi’s to induce apoptosis in human DLBCL, eight cell lines, from different DLBCL subtypes (see Materials and Methods), were treated with either 300 nM trichostatin A (TSA) [37] or 3 µM vorinostat [38], [39] for 24 h. Whole-cell extracts were then prepared, and cleavage of the caspase-3 substrate PARP was measured by Western blotting. As shown in Fig. 1, there was a range of PARP cleavage in these cell lines following treatment with either HDACi. SUDHL2, Pfeiffer, and SUDHL4 cells showed nearly complete cleavage of PARP. There was partial cleavage of PARP in BJAB, Farage, RC-K8, and SUDHL8 cell lines. In one cell line, SUDHL6, there was no detectable PARP cleavage following treatment with either HDACi.

### Trichostatin A and Vorinostat Induce Apoptosis in SUDHL4, but not in SUDHL6 Cells

**Figure 2:**
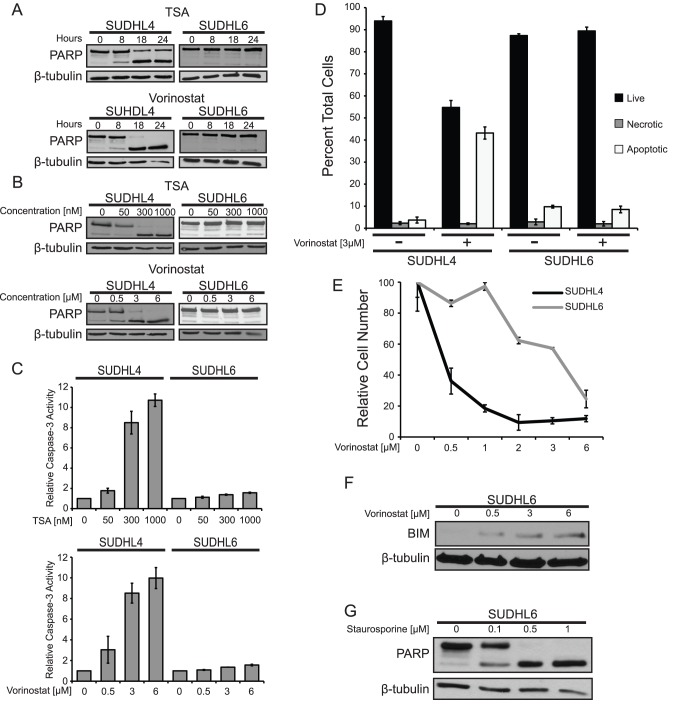
Trichostatin A and vorinostat induce apoptosis and inhibit growth in a time- and dose-dependent manner. (**A**) SUDHL4 and SUDHL6 cells were treated with either 300 nM TSA or 3 µM vorinostat for the indicated times. Whole-cell extracts were made, and Western blotting for PARP and β-tubulin (as a normalizing control) was performed. (**B**) SUDHL4 and SUDHL6 cells were treated for 14 h with the indicated concentrations of either TSA or vorinostat. PARP cleavage and β-tubulin levels (as a normalizing control) were assessed by Western blotting of whole-cell extracts. (**C**) Relative caspase-3 activity was measured in cells treated for 14 h with either TSA or vorinostat as indicated. The values represent the average relative fluorescence of three independent assays as compared to untreated samples (1.0). Error bars indicate standard error. (**D**) SUDHL4 and SUDHL6 cells were treated with 3 µM vorinostat for 14 h, cells were stained with acridine orange and ethidium bromide, and counted for live (black bars), necrotic (grey bars), and apoptotic (white bars) cells (see Materials and Methods). Each value is the average of three independent experiments and error bars indicate standard error. (**E**) Growth inhibition by vorinostat was assessed by treating SUDHL4 and SUDHL6 cells with increasing concentrations of vorinostat for 72 h, and cells were then counted using a hemocytometer. The relative numbers of cells are percentages as compared to cells grown for the same amount of time in the absence of vorinostat. The results are the averages of three separate treatment samples. Error bars indicate standard deviation. (**F**) SUDHL6 cells were treated with the indicated concentrations of vorinostat for 4 h. BIM and β-tubulin (as a normalizing control) were detected by Western blotting of whole-cell extracts. (**G**) SUDHL6 cells were treated for 24 h with the indicated concentrations of staurosporine. PARP cleavage and β-tubulin (as a normalizing control) were assessed by Western blotting of whole-cell extracts.

To further investigate the ability of TSA and vorinostat to induce apoptosis in a sensitive vs. a resistant DLBCL cell line (SUDHL4 vs. SUDHL6, respectively), cells were treated with 300 nM TSA or 3 µM vorinostat for increasing times up to 24 h and PARP cleavage was measured. Following treatment with either drug, SUDHL4 cells showed a time-dependent increase in PARP cleavage, whereas SUDHL6 cells showed no significant change in PARP cleavage, even at 24 h (Fig. 2A). Similarly, both TSA and vorinostat induced PARP cleavage and caspase-3 activity in a dose-dependent manner in SUDHL4 cells, but not in SUDHL6 cells (Figs. 2B and 2C). Additionally, SUDHL4 cells treated with 3 µM vorinostat for 14 h showed an approximately 40% increase in apoptotic nuclei compared to untreated cells, as judged by acridine orange and ethidium bromide staining; SUDHL6 cell showed no increase in apoptotic nuclei (Fig. 2D). Of note, there was no change in necrotic nuclei in either cell line, confirming that the cell death induced by vorinostat in SUDHL4 cells is apoptosis.

We next assessed the effect of vorinostat on cell proliferation by treating SUDHL4 and SUDHL6 cells with increasing concentrations for 72 h, and then comparing the number of cells to control, untreated plates (Fig. 2E). SUDHL4 cells were quite sensitive to inhibition of cell proliferation by vorinostat, showing an IC_50_ of less than 0.5 µM, whereas the IC_50_ for SUDHL6 cells was approximately 6-fold higher (∼3 µM). Thus, similar to what was seen with vorinostat-induced apoptosis, SUDHL4 cells are more sensitive to cell growth inhibition by vorinostat than SUDHL6 cells. The concentrations of vorinostat that inhibited cell proliferation in both SUDHL4 and SUDHL6 cells were well below the concentrations required to induce apoptosis in the respective cell lines (i.e., as in Figs. 2A–D). We have obtained similar results in these cell lines with other apoptosis-inducing compounds [36], indicating that cellular events that lead to cell proliferation arrest generally take place at lower concentrations than are required to induce apoptosis.

HDACi treatment has been shown to up-regulate expression of the pro-apoptotic BH3-only protein BIM in human colon carcinoma, osteosarcoma, leukemia, and multiple myeloma cell lines [27], [40]. To determine whether BIM expression can be induced by vorinostat in HDACi-resistant SUDHL6 cells, SUDHL6 cells were treated with increasing concentrations (0.5, 3, and 6 µM) of vorinostat for 4 h, and BIM Western blots were performed on whole-cell extracts. Treatment with vorinostat increased expression of BIM in a dose-dependent fashion as compared to untreated SUDHL6 cells (Fig. 2F). Therefore, the resistance of SUDHL6 cells to vorinostat-induced apoptosis does not appear to be due to an inability of BIM to be induced by vorinostat in these cells. Furthermore, the HDACi-resistant cell line SUDHL6 can be induced to undergo apoptosis by staurosporine (Fig. 2G), a general kinase inhibitor that is a potent inducer of caspase-mediated apoptosis. Thus, SUDHL6 cells do not have a general resistance to apoptosis-inducing agents.

### Over-expression of BCL-2, BCL-XL, and BIM Can Affect the Sensitivity of DLBCL Cell Lines to HDACi-induced Apoptosis

TSA and vorinostat have been shown to induce apoptosis through a BCL-2/caspase-3-dependent mechanism in a number of cell types including mammary carcinomas [40], retinoblastoma [41], and leukemic T cells [42]. Therefore, we hypothesized that shifting the overall balance of the BCL-2 family proteins to favor an anti- or pro-apoptotic expression profile would be sufficient to alter a cell’s resistance or sensitivity, respectively, to an apoptosis-inducing HDACi [5], [7], [13], [15], [18], [20], [23]–[29]. To that end, we modulated the dynamics of the BCL-2 protein family levels by over-expressing a relevant anti- or pro-apoptotic protein and then assessing the corresponding change in DLBCL sensitivity to vorinostat and TSA.

**Figure 3:**
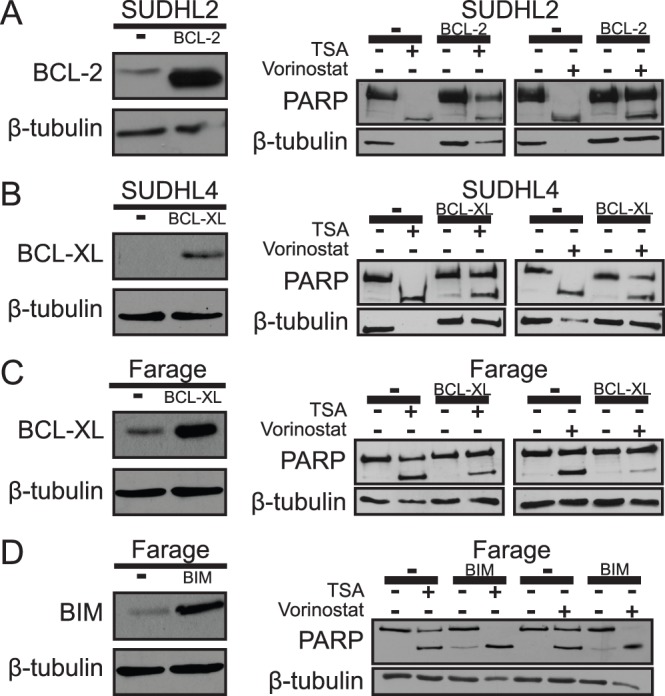
HDACi sensitivity is decreased by over-expression of BCL-2 or BCL-XL and increased by over-expression of BIM. Creation of four independent cell lines (SUDHL2 cells over-expressing BCL-2, SUDHL4 cells over-expressing BCL-XL, Farage cells over-expressing BCL-XL, and Farage cells over-expressing BIM) was confirmed by Western blotting for BCL-2, BCL-XL, and BIM (**A–D**) and compared to empty vector (–) transduced cells. β-tubulin was used as a normalizing control. (**A**) SUDHL2-BCL-2, (**B**) SUDHL4-BCL-XL, (**C**) Farage-BCL-XL, and (**D**) Farage-BIM cell lines were treated with either 300 nM TSA or 3 µM vorinostat for 24 h. Whole-cell extracts were made and Western blotting for PARP cleavage and β-tubulin (as a normalizing control) was performed. All cells over-expressing the protein of interest were compared to empty vector (–) transduced control cells.

To determine whether BCL-2 could protect sensitive cells from HDACi-induced apoptosis, SUDHL2 cells were infected with a retroviral vector that directed increased expression of mouse BCL-2, which is slightly smaller than the endogenous human BCL-2 in these cells (Fig. 3A) [36]. Control and SUDHL2-BCL-2 cells were treated with TSA or vorinostat, and extracts were probed for PARP cleavage (Fig. 3A). TSA and vorinostat both induced less PARP cleavage in SUDHL2-BCL-2 cells as compared to control cells (Fig. 3A, Table S1), indicating that ectopic expression of BCL-2 has an anti-apoptotic effect on these cells.

We next wanted to determine whether over-expression of pro-apoptotic BCL-XL could also render DLBCL cells less sensitive to HDACi treatment. Therefore, we infected SUDHL4 and Farage cells (which express moderately low levels of BCL-XL) with a retroviral vector encoding BCL-XL or with the empty vector. Pools of infected cells were selected with puromycin and Western blotting confirmed over-expression of BCL-XL in these retrovirally transduced cell lines (Fig. 3B and 3C). To compare their relative sensitivity to HDACi-induced apoptosis, control and BCL-XL-over-expressing cells were treated with 300 nM TSA or 3 µM vorinostat for 24 h and extracts were probed for PARP cleavage (Figs. 3B and 3C). In both SUDHL4 and Farage cells, over-expression of BCL-XL reduced PARP cleavage in response to treatment with TSA or vorinostat (Figs. 3B and 3C; Table S1), indicating that over-expression of BCL-XL has a protective effect on these cells.

We next determined whether Farage cells, which express moderately low amounts of pro-apoptotic BIM, could be made more sensitive to HDACi treatment by over-expression of BIM. Thus, Farage cells were infected with a retroviral vector that directed increased expression of BIM (Fig. 3D). As above, control and Farage-BIM cells were then treated with TSA or vorinostat and extracts were probed for PARP cleavage (Fig. 3D). TSA and vorinostat both induced more PARP cleavage in Farage-BIM cells as compared to control Farage cells (Fig. 3D, Table S1), indicating that over-expression of BIM sensitizes these cells to HDACi’s.

### Pharmacological Modulation of BCL-2 Family Member Interactions Can Sensitize DLBCL Cell Lines to Vorinostat-induced Apoptosis

**Figure 4:**
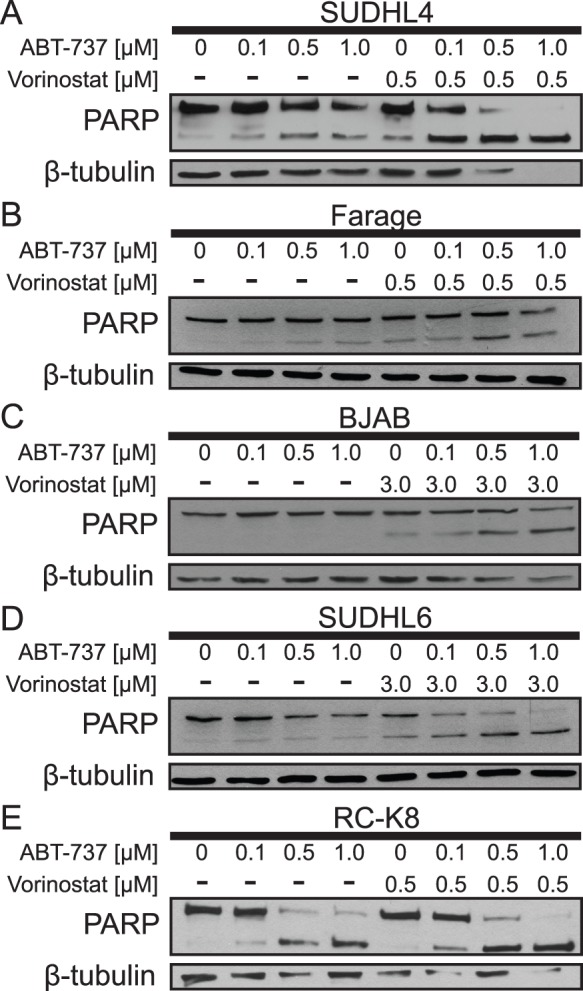
Vorinostat and the BH3 mimetic ABT-737 cooperate to increase apoptosis in DLBCL cell lines. (**A**) SUDHL4, (**B**) Farage, (**C**) BJAB, (**D**) SUDHL6, and (**E**) RC-K8 cells were treated with ABT-737 in a dose-dependent manner with and without a sub-optimal level (0.5 µM) or a higher concentration (3 µM) of vorinostat. Whole-cell extracts were subjected to Western blotting to assess PARP cleavage and β-tubulin levels (as a normalizing control).

ABT-737 is a BH3 mimetic that disrupts the interaction of BCL-2 and BCL-XL with BH3-containing pro-apoptotic proteins, such as BIM, NOXA, and BMF [7], [27], [28]. Based on our results above and similar studies [7], we reasoned that ABT-737 would sensitize DLBCL cell lines to HDACi-induced apoptosis. As shown in Fig. 4, treatment with up to 1 µM ABT-737 alone for 24 h induced little or no PARP cleavage in SUDHL4, Farage, BJAB, or SUDHL6 cells (Figs. 4A–D, respectively). However, as judged by PARP cleavage, treatment with ABT-737 sensitized all four of these cell lines to vorinostat-induced apoptosis. In SUDHL4 and Farage cells, ABT-737 increased their sensitivity to suboptimal doses of vorinostat (0.5 µM) (Figs. 4A and 4B), whereas in BJAB and SUDHL6 cells (Figs. 4C and 4D, respectively), ABT-737 increased PARP cleavage only with higher doses of vorinostat (3 µM). Of note, one cell line, RC-K8, showed a high level of PARP cleavage with ABT-737 alone (Fig. 4E). Taken together, these data show that combined HDACi/ABT-737 treatment is more effective than either agent alone at inducing apoptosis in many DLBCL cell lines. In particular, the HDACi-resistant cell line SUDHL6 was sensitive to apoptosis induced by combined treatment with HDACi/ABT-737 under conditions where neither agent alone induced apoptosis. The ability of ABT-737 to sensitize DLBCL cell lines to HDACi’s implicates BCL-2 family interactions as a factor in the cell’s response to HDACi treatment. It is likely a balance of pro- and anti-apoptotic BCL-2 protein expression that governs sensitivity to HDACi’s [16]. Of note, expression of no single BCL-2 family member, caspase, or common DLBCL marker protein could be correlated with HDACi sensitivity (Figs. S1 and S2), suggesting that DBLCL subtype per se is not relevant to HDACi sensitivity.

### Isolation of SUDHL4 Cell Lines with Reduced Sensitivity to Vorinostat

In an effort to isolate variants with decreased sensitivity to vorinostat, SUDHL4 cells were treated repeatedly with increasing concentrations of vorinostat (see Materials and Methods). SUDHL4 vorinostat-resistant (SUDHL-VR) cells could tolerate up to 3 µM vorinostat for 48 h, but after that, the cultures did not survive. SUDHL-VR cells were then cultured in media without vorinostat so that they could be compared to parental SUDHL4 cells for vorinostat-induced apoptosis and proliferation arrest.

**Figure 5:**
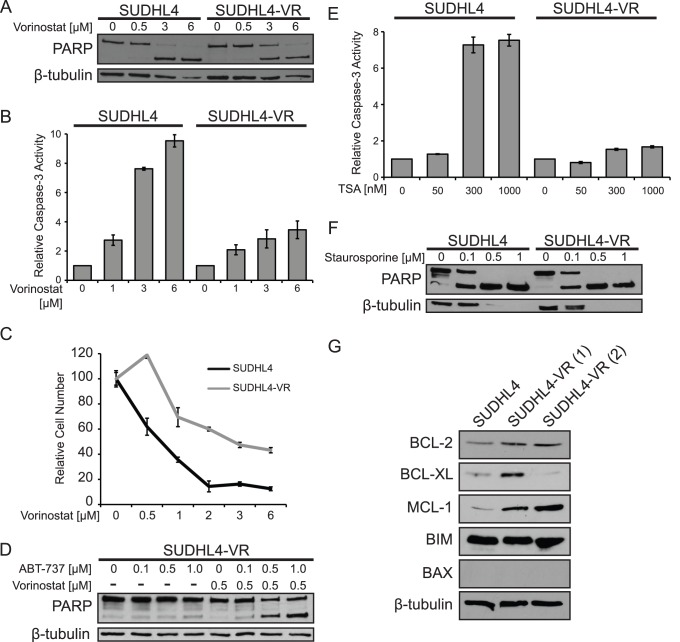
SUDHL4 cells selected for vorinostat resistance show decreased sensitivity to vorinostat-induced apoptosis and cell proliferation inhibition. (**A**) SUDHL4 and SUDHL4-VR cells were treated for 24 h with the indicated concentrations of vorinostat. PARP cleavage and β-tubulin (as a normalizing control) were assessed by Western blotting of whole-cell extracts. (**B**) Relative caspase-3 activity was measured in cells treated for 14 h with the indicated concentrations of vorinostat. The values represent the average relative fluorescence as compared to untreated samples (1.0) from three independent assays. Error bars indicate standard error. (**C**) Growth inhibition by vorinostat was assessed by treating SUDHL4-VR cells with increasing amounts of vorinostat for 72 h, and then counting cells using a hemocytometer. The relative numbers of cells are percentages as compared to cells incubated for the same amount of time in the absence of vorinostat. The results are the averages of three separate treatment samples. Error bars indicate standard deviation. (**D**) SUDHL4-VR cells were treated with the indicated concentrations of ABT-737 with and without a sub-optimal level (0.5 µM) of vorinostat. Whole-cell extracts were subjected to Western blotting for PARP cleavage and β-tubulin levels (as a normalizing control). (**E**) Relative caspase-3 activity was measured in cells treated for 14 h with TSA as indicated. The values represent the average relative fluorescence of three independent assays as compared to untreated samples. Error bars indicate standard error. (**F**) Cells were treated for 24 h with the indicated concentrations of staurosporine. PARP cleavage and β-tubulin (as a normalizing control) were assessed by Western blotting of whole-cell extracts. (**G**) Western blotting for the indicated BCL-2 family members in parental SUDHL4 cells and in two independent populations of SUDHL4-VR cells (1 and 2). β-tubulin was used as a protein loading control. The SUDHL4-VR(1) cell line was used for experiments in panels A–F.

To compare their sensitivity to vorinostat-induced apoptosis one of the SUDHL4-VR cell lines (clone 1) and parental SUDHL4 cells were both treated with increasing amounts of vorinostat for 24 h and then PARP cleavage was assessed by Western blotting. At 3 and 6 µM vorinostat, SUDHL4-VR cells displayed reduced PARP cleavage (Fig. 5A) and reduced caspase-3 activity (Fig. 5B) as compared to control SUDHL4 cells. Additionally, as compared to parental SUDHL4 cells, SUDHL4-VR cells were less sensitive to vorinostat-induced inhibition of cell proliferation (Fig. 5C). When treated with increasing concentrations of ABT-737, SUDHL4-VR cells showed nearly complete resistance to ABT-737 alone and only moderate PARP cleavage at high doses of ABT-737 in combination with low levels of vorinostat (Fig. 5D, compare to Fig. 4A). As compared to parental SUDHL4 cells, SUDHL4-VR cells were also less sensitive to TSA-induced apoptosis (Fig. 5E). However, staurosporine induced dose-dependent PARP cleavage upon treatment for 24 h (Fig. 5F), indicating that caspase-dependent apoptosis is not generally impaired in SUDHL4-VR cells.

To investigate the mechanism by which SUDHL4-VR cells develop resistance to vorinostat, the two independent isolates of SUDHL4-VR cells were compared to SUDHL4 cells for expression of the BCL-2 family members BCL-2, BCL-XL, MCL-1, BIM, and BAX (Fig. 5G). Among these proteins, expression of at least two of the anti-apoptotic proteins BCL-2, BCL-XL, and MCL-1 were increased in SUDHL4-VR cells: that is, expression of anti-apoptotic BCL-2 family proteins was increased in SUDHL4-VR cells as compared to the parental SUDHL4 cells, indicating that these cells might be more resistant to vorinostat-induced apoptosis due to elevated levels of BCL-2, BCL-XL, and/or MCL-1.

## Discussion

Our results show for the first time that vorinostat and the BH3 mimetic ABT-737 can cooperate to induce cell death in DLBCL in a BCL-2 family protein-dependent manner, a finding that is similar to the effects of these compounds in other types of cancer [5]–[7], [13], [25], [27], [29]–[34], [43], [44]. We also show that DLBCL cell lines can be made resistant to vorinostat, which may be useful in understanding the molecular mechanism by which patients develop resistance to HDACi treatment in the clinic.

HDACi’s are currently being used to treat a variety of lymphomas and leukemias [5]–[7], and some of these studies, as well as the results we report here, indicate that the levels of pro- or anti-apoptotic BCL-2 family proteins may be important for determining sensitivity to HDACi treatment. The emerging model for BCL-2-dependent regulation of apoptosis, as reviewed by Walensky and Gavathiotis [16], proposes that anti-apoptotic proteins (such as BCL-2 and BCL-XL) neutralize BH3-containing apoptotic modulators and pro-apoptotic activator proteins, while BH3-only proteins can also neutralize anti-apoptotic proteins. Agents that can increase the levels of free pro-apoptotic BH3-containing proteins can thus decrease the threshold needed to induce apoptosis. To overcome the complex BCL-2 protein regulatory network, HDACi’s have been combined with the BH3 mimetic ABT-737 to release BH3-containing modulator and pro-apoptotic activator proteins from sequestration by anti-apoptotic BCL-2 proteins [14], [15]. Although we found that BIM expression is increased rapidly following vorinostat treatment in the HDACi -resistant SUDHL6 cell line (Fig. 2F), it is important to note that SUDHL6 cells have levels of constitutive BIM that are more than 10-fold lover than HDACi-sensitive SUDHL4 cells (see Fig. S1). Therefore, HDACi-induced levels of BIM in SUDHL6 cells are by comparison still quite low. Additionally, SUDHL6 cells can be induced to undergo apoptosis when treated with a combination of vorinostat and ABT-737. These results suggest that even though BIM is up-regulated by HDACi treatment in SUDHL6 cells, it is not to a high enough level to overcome protective sequestration of BIM by BCL-2 and BCL-XL, a situation which can be overcome by releasing BIM or other apoptosis-sensitizing proteins from their binding pockets by treatment with ABT-737. Moreover, one study [8] reported that SUDHL6 cells show a significant accumulation of apoptotic cells after treatment with 5 µM vorinostat for 96 h, a time and concentration much greater than we have used here.

Ectopic over-expression of BIM in Farage cells increased their sensitivity to HDACi-induced apoptosis (Fig. 3D), consistent with BIM’s emerging status as a key pro-apoptotic regulator of cell survival. Despite repeated attempts, we have not been able to isolate RC-K8 cells that over-express BIM, likely because BIM over-expression is, in and of itself, toxic in certain cell types. Of note, RC-K8 cells have almost no basal expression of BIM (Fig. S1), which might, in general, make them quite sensitive to artificial increases in BIM expression.

We have also created two independent DLBCL cell lines (SUDHL4-VR) with reduced sensitivity to vorinostat. In both SUDHL4-VR cell lines, MCL-1 expression was increased as compared to parental cells, and in one of the VR cell lines, BCL-XL expression was also increased. Additionally, there is a slight increase in BCL-2 expression in both SUDHL4-VR cell lines. Expression of these anti-apoptotic proteins would be expected to shift the balance of the BCL-2 family towards an anti-apoptotic profile. It is reasonable to hypothesize that the increased levels of these anti-apoptotic proteins would sequester more BIM and BAX, making SUDHL-VR cells less susceptible to vorinostat-induced apoptosis. Of note, BCL-XL can be transcriptionally regulated by the NF-κB, JAK/STAT, AP-1, and Ets transcription factors [45], [46], MCL-1 is a transcriptional target of STAT3 and both are often up-regulated in aggressive lymphomas [47]–[51], and BCL-2 is routinely associated with the ABC DLBCL subtype and increased NF-κB activity, and has been shown to be a transcriptional target of STAT3 [47], [52]–[55]. Considering that HDACi treatment results in deregulated STAT and NF-κB signaling [56]–[59] and that some lymphoma cells that are less sensitive to HDACi treatment have elevated levels of JAK/STAT activity [60], it is not surprising that an HDACi-sensitive cell line (SUDHL4) might up-regulate expression of any number of the anti-apoptotic proteins BCL-XL, BCL-2, and/or MCL-1 in response to prolonged and repetitive exposure to HDACi treatment. Our in vitro finding with SUDHL4-VR cells suggests that similar tumor cell resistance could occur in DLBCL patients treated with repeated doses of vorinostat. Acquired resistance to ABT-737 has also been observed in DLBCL cell lines, including SUDHL4 cells, which was attributed to an up-regulation in MCL-1 [44], [61]. ABT-737, which displaces BH3-containing BCL-2 family proteins from the BH3-binding pocket in BCL-2 and BCL-XL but not MCL-1 [14], [26], and has been shown to induce apoptosis in MCL-1-expressing cells only when MCL-1 is down-regulated [62], [63] indicating that BCL-2 inhibitors that also target MCL-1 might have clinical importance [64].

### Conclusions

The goal of this research was to identify the molecular mechanism that regulates sensitivity to HDACi treatment in DLBCL. Our results show that DLBCL cells exhibit a range of HDACi-sensitivity that can be regulated by the BCL-2 family. Specifically, ectopic expression of BCL-2 family members modulates HDACi-sensitivity, while induced HDACi resistance leads to increased expression of the anti-apoptotic proteins such as BCL-XL, BCL-2, and MCL-1. Overall, our results suggest that HDACi-resistant DLBCL patients may benefit from combination therapy utilizing HDACi- and ABT-737-like compounds.

## Materials and Methods

### Cell Culture

A293T human embryonic kidney cells were cultured in Dulbecco’s Modified Eagle’s Medium (DMEM) supplemented with 10% heat-inactivated fetal bovine serum (FBS) (Biologos, Montgomery, IL, USA) as previously described [65]. Human DLBCL cell lines were cultured in DMEM or RPMI supplemented with 10–20% heat-inactivated FBS. DLBCL cell lines used in this study are as follows: BJAB (GCB-DLBCL), RC-K8 (ABC-DLBCL), and SUDHL4 (GCB-DLBCL) [65]; Pfeiffer (GCB-DLBCL) [66] and Farage (GCB-DLBCL) [67] (gifts of Dr. Gerald Denis, Boston University School of Medicine); SUDHL2 (ABC-DLBCL) and SUDHL8 (GCB-DLBCL) (gifts of Dr. Riccardo Dalla-Favera, Columbia University [68]); and SUDHL6 (GCB-DLBCL) [35].

The retroviral vectors pMSCV, pMSCV-BCL-XL, and pMSCV-BCL-2 were described previously [36], [69]. pMSCV-BIM was created by PCR amplifying the human BIM cDNA with forward (5′GCATAGATCTATGGCAAAGCAACCTTCTGATG3’) and reverse (5′GCATGAATTCTCAATGCATTCTCCACACCAG3’), and subcloned into BglII/EcoRI-digested pMSCV.

Virus stocks for pMSCV-based vectors were generated using the helper plasmid pcL10a1, as described previously [54], [69]. Briefly, A293T cells were transfected using polyethylenimine (PEI) (Polysciences, Warrington, PA, USA) at a plasmid DNA:PEI ratio of 1∶3 in DMEM containing 10% FBS. The next day, the transfection media was replaced with fresh DMEM containing 10% FBS, and virus was harvested two and three days after transfection. Two ml of virus (in the presence of 8 µg/ml polybrene) was used to infect 10^6^ cells using the spin infection method [54], [65]. Two days after infection, cells were selected with 2 µg/ml puromycin (Sigma Aldrich, St, Lous, MO, USA).

### Treatment of Cells with Trichostatin A, Vorinostat, and/or ABT-737

For all treatment experiments, except for acridine orange and ethidium bromide staining (see below), 10^6^ cells were plated in 2 ml of media and allowed to grow for 6 h. Trichostatin A (Sigma Aldrich), vorinostat (Cayman Chemical, Ann Arbor, MI, USA), and/or ABT-737 (Active Biochemicals Co., Ltd., Hong Kong) was then added directly to the cultures, and cells were incubated for the indicated times.

Selection of SUDHL4-VR cells was performed by treating parental SUDHL4 cells with 1 µM vorinostat for 24 h and then allowing the cells to recover in the absence of drug. When the cells repopulated the dish, they were treated with 1 µM vorinostat for 48 h and allowed to recover. These cells were then treated with 1 µM vorinostat for 72 h and allowed to recover. Once the cells repopulated the dish, the same dosing cycle was performed with 2 µM and then 3 µM vorinostat. For each treatment, a fraction of the recovered cells were grown in the absence of vorinostat, representing the most recent vorinostat-resistant population, in case the current treatment was toxic to the cells. SUDHL4-VR cells survived up to 3 µM for 48 h and this population was used for studies in this paper.

### Western Blotting

Western blotting was performed as described [36], [65]. Whole-cell extracts were prepared in AT buffer (20 mM HEPES, pH 7.9, 1 mM EDTA, 1 mM EGTA, 20 mM Na_4_P_2_O_7_, 1 mM DTT, 1% v/v Triton X-100, 20% w/v glycerol, 1 mM Na_3_VO_4_, 1 µg/ml PMSF, 1 µg/ml leupeptin, 1 µg/ml pepstatin). Samples containing equal amounts of protein were separated on SDS-polyacrylamide gels and transferred to nitrocellulose membranes (Micron Separation Inc., Westborough, MA, USA). Antibodies were as follows: BCL-2 (1∶500, BD Transduction Laboratories, San Jose, CA, USA, #610538), BCL-XL (1∶1000, Cell Signaling Technology, Danvers, MA, USA #2762), MCL-1 (1∶500, Santa Cruz Biotechnology, Santa Cruz, CA, USA, #819), BIM (1∶1000, Cell Signaling Technology, #2819), BAX (1∶500, Cell Signaling Technology), HRK (1∶1000, ProSci, Poway, CA, USA, #3771), BID (1∶1000, Santa Cruz Biotechnology, #11423), Caspase-3 (1∶500, Santa Cruz Biotechnology, #7148), Caspase-8 (1∶500, Santa Cruz Biotechnology, #7890), c-REL (1∶10000, kind gift of Nancy Rice, #265), p65 (1∶2000, kind gift of Nancy Rice, #1226), BCL6 (1∶1000, Cell Signaling Technology, #4242S), CD10 (1∶500, Santa Cruz Biotechnology, #58939), PARP (1∶500, Santa Cruz Biotechnology, #1750), and β-tubulin (1∶500, Santa Cruz Biotechnology, #9104). Nitrocellulose filters were incubated with primary antiserum for 1 or 18 h at room temperature or 4°C, respectively. The appropriate horseradish peroxidase-labeled secondary antiserum was added and immunoreactive proteins were detected with the SuperSignal Dura West Extended Duration Substrate chemiluminescence detection system (ThermoFisher Scientific, Waltham, MA, USA).

### Caspase-3 Activity Assay

Caspase-3 activity was determined as described previously [36]. Briefly, cells treated with either TSA or vorinostat for the indicated times were lysed by three freeze-thaw cycles. Equal amounts of cell extracts were incubated in reaction buffer containing 50 µM of the fluorogenic substrate Ac-DEVD-AMC (Biomol Research Labs, Plymouth Meeting, PA, USA) for 1 h at 37°C. Caspase-3 activity was measured using a Victor^3^ Multilabel Plate Reader model 1420 (Perkin Elmer, Waltham, MA, USA) using an excitation of 380 nm and measuring an emission of 460 nm. The assay was performed three times and values represent the average fluorescence of three independent assays as compared to untreated control samples.

### Acridine Orange and Ethidium Bromide Staining for Cell Death

Acridine orange and ethidium bromide staining for apoptosis was performed as previously described [70]. Briefly, 2.5 × 10^5^ cells were treated with 3 µM vorinostat for 14 h. Cells were then treated with a 100 µg/ml acridine orange/ethidium bromide mixture and more than 130 cells per field were visualized with an Olympus 1×70 microscope and counted.

### Cell Proliferation Assay

Briefly, 2.5×10^5^ cells were treated with the indicated concentrations of vorinostat for 72 h, and cells were then counted using a hemocytometer. The number of cells in vorinostat-treated cultures is reported as a percentage of the number of cells in untreated cells grown for the same amount of time. The results are the averages of three separate treatment samples. Error bars indicate standard deviation.

## Supporting Information

Figure S1 **Expression of individual BCL-2 family members does not correlate with HDACi sensitivity in DLBCL cell lines.** Eight DLBCL cell lines were probed for expression of the anti-apoptotic proteins BCL-2, BCL-XL, and MCL-1, the apoptosis sensitizers BIM, HRK, and BID, and the pro-apoptotic activator BAX. Also included in this screen were effector caspase-3 and -8 and β-tubulin (as a loading control).
(EPS)

Figure S2 **Expression of common DLBCL protein markers does not correlate with HDACi sensitivity in DLBCL cell lines.** Western Blotting was performed for the indicated protiens in eight DLBCL cell lines. β-tubulin was used as a loading control.
(EPS)

Table S1 **Quantification of the extent of PARP cleavage in** **Fig. 3** **.** HDACi-induced PARP cleavage is decreased by over-expression of BCL-2 or BCL-XL and increased by over-expression of BIM. The numbers represent the percentage of HDACi-induced PARP cleavage as quantified from the bands in the Western blots in Figure 3 using ImageJ. Breifly, band intensities of cleaved and uncleaved PARP were added together to give the total PARP for each sample. The percentage of cleaved PARP was then calculated by dividing the value of the cleaved PARP (lower band) by the total PARP.
(EPS)
